# The Attitudes of Children Undergoing Orthodontic Treatment toward Face Mask Wearing during the COVID-19 Pandemic: A Cross Sectional Study

**DOI:** 10.3390/children9070989

**Published:** 2022-07-01

**Authors:** Jessica Olivia Cherecheș, Gabriela Ciavoi, Abel Emanuel Moca, Raluca Iurcov, Raluca Dima, Marius Bembea, Luminița Ligia Vaida

**Affiliations:** 1 Department of Dentistry, Faculty of Medicine and Pharmacy, University of Oradea, 10 Piața 1 Decembrie Street, 410073 Oradea, Romania; chereches_jessica@yahoo.com (J.O.C.); riurcov@uoradea.ro (R.I.); razdima@gmail.com (R.D.); ligia_vaida@yahoo.com (L.L.V.); 2 Department of Preclinical Disciplines, Faculty of Medicine and Pharmacy, University of Oradea, 10 Piața 1 Decembrie Street, 410073 Oradea, Romania; bembea13@yahoo.com

**Keywords:** children, orthodontics, face mask, COVID-19

## Abstract

During the COVID-19 pandemic, the protective face mask has proven to be essential. The protective face masks cover the lower part of the face, including teeth and, for orthodontic patients, the orthodontic appliances. The aim of this study was to assess the impact that the restrictive measures that were imposed during the COVID-19 pandemic and, especially, wearing a protective face mask had on a sample of Romanian children, and to compare the results previously obtained on a sample of Romanian teenagers with the results obtained after investigating children under the age of 12 years. The cross-sectional survey was conducted in two orthodontic offices from the city of Oradea, Romania. The study sample included children with ages between 8 and 11.9 years that were undergoing an orthodontic treatment with removable or fixed orthodontic appliances. After obtaining the results, comparisons were made with the answers provided by a group of adolescents previously investigated. The questionnaires consisted of 9 items that investigated children’ attitudes toward protective face mask wearing and other aspects related to the COVID-19 pandemic. Two hundred fifty-six children were included in the study (53.1% female patients, 46.9% male patients). Most of the children were not worried that face masks would hide their orthodontic appliances (Item 1—Never, 40.2%; Rarely, 28.9%) and did not consider that the necessity of face mask wearing negatively impacted their desire to undergo an orthodontic treatment, despite the fact that it covered the appliances (Item 2—Never, 37.1%; Rarely, 31.6%). However, 44.5% of children were not happy because they had to wear a face mask during the orthodontic treatment, considering the fact that it covered the orthodontic appliance (Item 6), and most patients (49.2%) did not want the face mask to continue to be mandatory (Item 7). Although children were not happy that they had to wear a face mask that covered the orthodontic appliances, protective face masks were generally well tolerated by Romanian children.

## 1. Introduction

At the end of 2019 in Wuhan, China, a new form of viral pneumonia was identified. It was caused by a coronavirus, SARS-CoV-2, and it was associated with a severe acute respiratory syndrome [1,2,3]. The initially local epidemic [2] rapidly spread in other countries, causing a public health problem worldwide [4]. For this reason, the World Health Organization issued an international warning on public health problems on 30 January 2020 [5]. COVID-19 symptoms are numerous, including neurological manifestations, such as headache, dizziness, myalgia and anosmia [6], as well as fatigue, fever, cough, dyspnea and diarrhea [7]. Signs of lung damage are frequently identified on radiographs or CT scans [8].

COVID-19 has a high rate of contagion and is transmitted from person to person [9], the incubation period varying between 2 and 14 days, with an average of 5 days [10]. The virus is transmitted by drops or aerosols resulting from speech, coughing or sneezing [11] from symptomatic or asymptomatic individuals, or from people that are in the incubation period [12]. The virus attaches to the eye, nasal and oral mucosa [10].

With the rapid increase in the number of COVID-19 cases, on 11 March 2020, the World Health Organization declared a global COVID-19 pandemic, and therefore, in order to reduce contamination, quarantine was recommended around the globe [13]. A number of restrictions were imposed, varying from country to country, and generally consisted of wearing a face mask, diligent hand washing [13], population testing, social distancing and lockdown [14]. Due to the impossibility to keep a distance of more than 1 m during dental treatments, dental professionals are exposed to an increased risk of contamination, with many of the maneuvers generating aerosols [15]. During the COVID-19 lockdown, dental patients were deeply affected. Routine dental treatments could no longer be performed or continued, except for dental emergencies, such as severe pain, infection, bleeding or trauma [16].

A particular group of patients who were affected by the discontinuation of the activity of dental offices, were patients undergoing an orthodontic treatment. Orthodontic treatment is frequently recommended for functional restoration and facial aesthetics improvement [17], and extends over longer periods of time, sometimes up to 24 months or even longer depending on the age and clinical condition of the patients [18]. It requires regular check-ups every 6 to 8 weeks [19]. Due to the lockdown, these check-ups were postponed [20], which led to extended periods of time required for finishing the orthodontic treatment [19], but also to complications and accidents [20]. The COVID-19 pandemic, due to the restrictions imposed, produced undesirable consequences on a mental, emotional and financial level [19], with patients becoming anxious and stressed as a result of the new situation [21].

The protective face mask has proven to be essential, and its wearing is recommended by the World Health Organization, starting at the age of 6 [22]. Despite parents’ initial concern about the possible harmful effects of wearing a face mask, studies have shown that both ventilation and oxygen intake have not been negatively affected [22]. The face mask covers the lower part of the face [23], including the teeth and, implicitly, the orthodontic appliances. Wearing a face mask could cause frustration due to the fact that the orthodontic appliances are no longer visible, as it is known that orthodontic appliances are often perceived as an elective luxury [24], and for children, the possibility of using colored elastic ligatures is one of the most appealing aspects during the orthodontic treatment [25].

In order to comprehend the impact of face mask wearing on adolescent orthodontic patients, with ages between 12 and 17.9 years, a study was conducted by the authors on a sample of Romanian adolescent orthodontic patients from Oradea, Romania [26]. The opinion of orthodontic patients under the age of 12, regarding face mask wearing and the suspension of dental offices’ activity was deemed necessary so that a wider range of patients would be investigated.

The aim of this study was to assess the impact that the restrictive measures that were imposed during the COVID-19 pandemic and, especially, wearing a protective face mask had on a sample of Romanian children, with ages between 8 and 11.9 years, undergoing orthodontic treatment with fixed or removable appliances. Another aim was to compare the results previously obtained on a sample of Romanian teenagers [26] with the results obtained after investigating children under the age of 12 years, and to discover whether children were more or less affected by face mask wearing and other restrictive measures.

## 2. Materials and Methods

### 2.1. Ethical Considerations

The research was carried out in conformity with the Helsinki Declaration of 1964 and its subsequent amendments. It was accepted by the Research Ethics Committee of the University of Oradea (No. 23 from 25 February 2021). All parents, legal guardians, and participants agreed to participate in this study before filling out the questionnaire.

### 2.2. Sample Size Calculation

GPower 3.1.9.7 software (Heinrich Heine University, Düsseldorf, Germany) was used to estimate the sample size. The study’s design took into account that the assessed items (which were presented in Likert scale style) would primarily be compared between age groups using Mann-Whitney U tests, and the appropriate grouping ratio should be 1:1. The minimum sample size was calculated to be 74 patients in each group (study group and control group) using a middle effect size of d = 0.5, a minimum power of 0.8, and =0.05 (a total of 148). The minimum total sample size for contingency tables should be 108, taking into account a medium effect size of w = 0.3 with Df = 2, a minimum power of 0.8, and =0.05. Using these numbers, it was calculated that the study would need a minimum of 74 individuals in each age group (for a total of 148 patients) to have a minimum power of 0.8 for the majority of the tests.

### 2.3. Participants and Data Collection

The study, which was conducted between February 2021 and July 2021, was planned as a cross-sectional survey and lasted six months. Strict restrictions, such as the requirement to wear a face mask and social distancing, were in effect at this time. The study was not preceded by a pilot study. However, a similar study, using the same items, was previously conducted on adolescents from Oradea, Romania.

The questionnaires consisted of 9 items that were specifically imagined for orthodontic patients. They were previously used on a sample of adolescent orthodontic patients from the city of Oradea, Romania, with ages between 12 and 17.9 years [26]. The printed questionnaires were applied in two private practices delivering orthodontic treatments from Oradea, NW Romania. For this research, they were handed to orthodontic patients ranging in age from 8 to 11.9 years old. The participants were patients undergoing an orthodontic treatment with fixed or removable appliances. All parents, caregivers, and patients were informed prior to completing the questionnaire that they were applied for research purposes and that by completing the questionnaires, they were confirming their agreement to anonymously take part in this study. Patients had the possibility to withdraw from the study at any time. No financial incentives were promised to the respondents, and no time limit was imposed. The language used for questionnaires was Romanian.

The study group consisted of patients that met the following inclusion criteria: orthodontic patients wearing a fixed orthodontic appliance (metallic or ceramic), bonded on the buccal surface of the teeth; orthodontic patients wearing a removable appliance during the night and day; all orthodontic appliances were visible in smile and speech; orthodontic patients with ages between 8 and 11.9 years; orthodontic patients living in Romania. The control group was represented by the orthodontic patients from the previously published article that investigated the attitudes of adolescent orthodontic patients toward face mask wearing during the COVID-19 pandemic, and it consisted of orthodontic patients with ages between 12 and 17.9 years old, who were wearing a fixed orthodontic appliance that was bonded on the buccal surface of teeth, and therefore it was visible in smile and speech, and were living in Romania [26]. 

Questionnaires that were incompletely or incorrectly filled out (more than one answer for the same Item) were excluded from this study.

**Figure 1:**
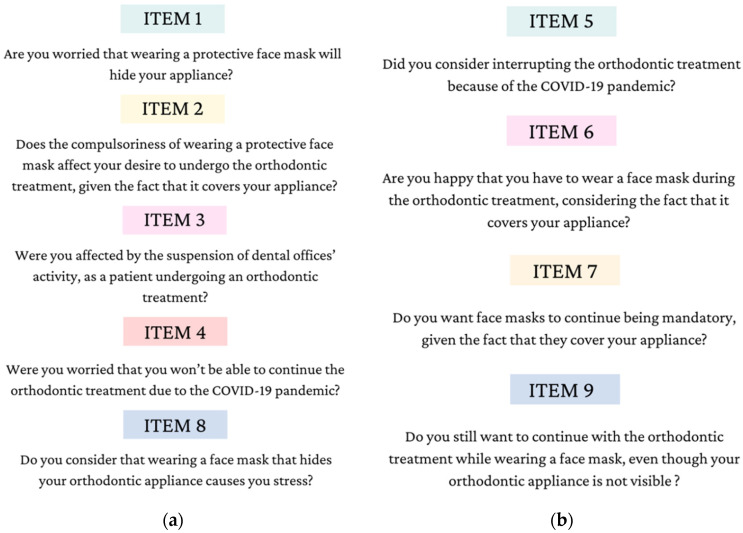
(**a**) Likert-type scale items; (**b**) three-option items [26].

For Items 1, 2, 3, 4, and 8, the authors used a Likert-type scale, with the following options: “never”, “rarely”, “occasionally”, “frequently” and “very frequently”. For items 5, 6, 7 and 9, there were three available options the respondents had to choose from, these being “no”, “yes” and “maybe” [26]. Items are translated and detailed in Figure 1.

### 2.4. Statistical Analysis

For the statistical analysis, IBMS SPSS Statistics 25 (IMB, Chicago, IL, USA), Microsoft Office Excel 2013 and Word 2013 (Microsoft, Redmond, WA, USA) were used. The Shapiro-Wilk test was used to determine the distribution of quantitative data, which were then expressed as medians with interpercentile ranges or means with standard deviations. The Mann-Whitney U test and the Kruskal-Wallis H test were used to examine independent quantitative variables having non-parametric distribution. The Spearman’s rho correlation coefficient was used to confirm the correlation between these two. Fisher’s Exact Test was used to test qualitative variables that were reported as absolute values or percentages. 

The data obtained after obtaining the contingency tables were detailed using Z-tests with Bonferroni correction. The results obtained after evaluating the independent quantitative variables were described using Dunn-Bonferroni tests.

## 3. Results

For the study group, the questionnaires were distributed to 285 children, undergoing an orthodontic treatment, of which 6 refused to participate. Out of the 279 questionnaires that were filled out, an additional 23 were excluded, with the final sample consisting of 256 survey forms filled out by 256 children.

For the control group, an initial number of 320 questionnaires were handed out, but 30 adolescents refused to participate. An additional number of 13 questionnaires were excluded, the final sample consisting of 277 survey forms filled out by 277 orthodontic patients [26].

### 3.1. Socio-Demographic Data

Data in Table 1 shows the distribution of the patients according to gender and living environment for the study group and the control group. Regarding age, the children included in the study group had a minimum age of 8 years and a maximum age of 11.9 years, with an average age of 9.89 years. The adolescents in the control group had a minimum age of 12 years and a maximum age of 17.9 years, with an average age of 14.91 years [26]. 

The results previously obtained for the control group by Cherecheș et al. (2022) [26] were used only to compare the answers provided by children and adolescents and were not repeated in detail. The results obtained for the study group will be presented in detail.

### 3.2. Children’s Attitudes toward Protective Face Mask Wearing and Other Restrictions Imposed during the COVID-19 Pandemic

The data in Table 2 show the distribution of patients according to the responses provided for the 9 items. Most patients included in the study were not at all worried that face masks would hide their appliances (Item 1). Most patients did not consider that the compulsoriness of face mask wearing affected their desire to undergo an orthodontic treatment, despite the fact that it covered the appliances (Item 2). Although most patients were not affected by the suspension of dental offices’ activity, a third of the respondents were affected by this aspect (Item 3). About half of the respondents did not consider interrupting the orthodontic treatment during the COVID-19 pandemic (Item 5). Nearly half (44.5%) of the patients were not happy because they had to wear a face mask during the orthodontic treatment, considering the fact that it covered the orthodontic appliance (Item 6), and most patients did not want the face mask to continue to be mandatory (Item 7).

### 3.3. Correlational Results

The gender and the living environment of the respondents were not statistically significantly correlated with any of the 9 items. Regarding the age of the patients, the only significant correlation was observed between the age of the patients and the answers provided for Item 1. The distribution of the variables was non-parametric according to the Shapiro-Wilk test (*p* < 0.05). The observed correlation was significant and negative, with a mild degree (*p* = 0.008), so that patients with a higher age had a significantly lower degree of concern about the fact that wearing a protective face mask would hide the orthodontic appliance compared to patients with lower ages. 

Statistically significant correlations were observed between the answers provided for Item 1 and Item 2, Item 1 and Item 8, Item 2 and Item 3 and Item 3 and Item 4. Thus, respondents who were more concerned about wearing a face mask that covered the orthodontic appliances stated significantly more frequently that they were more affected by the obligation to wear a protective face mask during the orthodontic treatment (Item 1 and Item 2). Respondents who were more affected by the obligation to wear a face mask during the orthodontic treatment stated that they were significantly more affected by the suspension of dental offices’ activity (Item 2 and Item 3). The correlative results are presented in Table 3.

### 3.4. Comparative Results

Comparisons were made according to the age of the respondents, their gender and living environment and the answers given for the different items. Regarding the age of the patients, significant differences were observed only between the age of the patients and the answers provided for Item 7. The age distribution was non-parametric in most groups according to the Shapiro-Wilk test (*p* < 0.05). The differences between the groups were statistically significant according to the Kruskal-Wallis H test (*p* = 0.006), and the post-hoc tests show that patients who wanted to wear a face mask had higher ages (median = 10.5 years, IQR = 9–11 years) compared to patients who stated that they did not want the face mask to be mandatory (median = 9 years, IQR = 9–11 years) (*p* = 0.007).

Regarding the gender of the patients, significant differences were observed between the gender of the patients and the answers provided for Item 4, so that male respondents felt significantly more worried about the possibility of not being able to continue the orthodontic treatment due to the COVID-19 pandemic compared to female respondents. Regarding the patients’ living environment, significant differences were identified between the patients’ living environment and the answers provided for Item 1 and Item 4. Respondents who lived in a rural area felt a significantly higher level of concern about wearing a protective face mask that would hide their appliance in comparison to patients who lived in urban areas, as well as a significantly higher level of concern about the possibility of not being able to continue the orthodontic treatment due to the COVID-19 pandemic than patients who lived in urban areas (Table 4).

Statistically significant differences were also observed between the answers provided for Item 3 and Item 5, as well as between Item 3 and Item 7. Thus, respondents who considered interrupting the orthodontic treatment due to the COVID-19 pandemic felt significantly less affected by the suspension of dental offices’ activity compared to patients who felt indecisive about interrupting the orthodontic treatment, and patients who did not want or wanted to continue to wear a face mask given the fact that they covered the orthodontic appliances were still significantly less affected by the suspension of dental offices’ activity compared to patients who were indecisive regarding the mandatory wearing of face masks (Table 5).

### 3.5. Comparisons between the Attitudes of Children (Study Group) and Adolescents (Control Group)

For the Likert-type scale items, significant differences were found between children and adolescents for Item 2 and Item 8. Thus, the adolescents in the control group felt less affected by the compulsoriness of face mask wearing during the orthodontic treatment compared to children in the study group (Item 2). Adolescents also felt significantly less stressed about wearing a face mask that covered the orthodontic appliance compared to the children in the study group (Item 8) (Table 6).

The data in Table 7 show the comparison between children and adolescents according to the answers provided for the 3-option items. Statistically significant differences were identified between children and adolescents for all 4 items. Children reported significantly more frequently that they considered interrupting the orthodontic treatment due to the COVID-19 pandemic, while adolescents reported significantly more frequently that they did not consider interrupting the orthodontic treatment (Item 5). Children stated significantly more often that they wanted to continue wearing a face mask, despite the fact that it covered the orthodontic appliance, while teenagers said significantly more often that they did not want to wear a face mask (Item 7). Children also stated significantly more often that they wanted to correct the position of their teeth despite the fact that they had to wear a protective face mask that covered the orthodontic appliance, while teenagers stated significantly more often that they did not want to correct the position of their teeth while wearing a face mask (Item 9).

## 4. Discussion

Dental malocclusion can be considered the result of a biological variation and requires orthodontic treatment [27]. The persistence of dental malocclusions in the absence of proper treatment negatively affects the quality of life of both young patients and their families [28] because it changes the dental and facial appearance [29]. Dental malocclusions are also responsible for issues related to mastication, phonation, swallowing [30] and traumatic occlusal contacts [31], which over time can cause periodontal damage [32,33].

Disharmonious facial appearance due to dental changes in children and adolescents is a very sensitive issue because it often attracts social discrimination with physical and psychological consequences [34]. At a social level, the way the physical aspect is perceived has a great influence on an individual’s self-esteem [35]. Signs of low self-esteem in patients with dental malocclusion are seen through anxiety, lack of social interaction and lack of communication, and in the absence of an early orthodontic treatment intended to correct dental malocclusions, these traumas persist even in adulthood [35,36]. Studies that assessed the attitudes of adolescents toward facial aesthetics showed that female patients were more affected by altered facial aesthetics compared to male patients and also showed a greater desire to initiate orthodontic treatment [37,38]. In the group of children that we investigated, male respondents felt significantly more worried than female respondents about the possibility of not being able to continue the orthodontic treatment due to the COVID-19 pandemic. This aspect could indicate that, in our study sample male patients were more affected by their facial appearance, and considered that COVID-19 pandemic could interrupt the process of dental and facial correction.

Impairment of dento-facial aesthetics, self-perception and social relationships are the main reasons for which parents go to the orthodontist with their children [39,40]. The improvement of facial aesthetics encourages them to accept the idea of wearing different orthodontic appliances [41] as a necessary mean for the improvement of the quality of life, [39,40] of the facial aspect and of the psychological status [42]. The beneficial role of the orthodontic treatment is easily seen in children and adolescents [43]. This could be the reason for which, when asked if they wanted to continue with the orthodontic treatment while wearing a face mask, even though the orthodontic appliances will no longer be visible, most of the children (69.5%) and adolescents (65.7%) answered positively. This could indicate that the final result of the orthodontic treatment is more important than the visibility of the orthodontic appliance for the patients investigated.

In the prevention and treatment of dental malocclusions, early therapy with a removable or fixed appliance ensures a therapeutic success in most cases [44]. There is a more frequent acceptance of metallic braces, among children and adolescents, due to the possibility of using colored elastic ligatures [45]. As proven by our experience, children and adolescents, seem to enjoy the fact that they wear orthodontic appliances and, quite often, are eager to show their smile during the orthodontic treatment. This is why we asked ourselves whether or not wearing a protective face mask that covers the lower part of the face, including the mouth and the orthodontic appliance, could affect their interest in wearing orthodontic appliances. Although most of the children investigated were not worried about wearing a face mask that covered the orthodontic appliance, 30.9% of them said that they were worried to some degree. In a similar way, 31.3% of all children investigated considered that their desire to undergo the orthodontic treatment was affected by the compulsoriness of face mask wearing. As such, for almost a third of all children that participated in this study, not having the orthodontic appliances visible in a smile and speech, represented a major disadvantage. These children should be motivated more by the parents and the orthodontists regarding the importance of the orthodontic treatment itself.

The COVID-19 pandemic brought great changes in the activity of dental offices, and later by suspending the activity of dental offices, doctors and patients faced an unprecedented situation [46]. The dental treatments could no longer be continued, and for the patients with orthodontic appliances, the treatments were significantly affected, considering the fact that they usually last up to 2 years and require regular check-ups. Item 3 investigated the respondents’ attitude toward the suspension of dental offices’ activity, as patients undergoing orthodontic treatments. Forty-three percent of all children were affected by this aspect to some degree. This warrants more interest toward the dental community and dental patients in the event of a new pandemic.

We chose to conduct the research in the form of a questionnaire because it is a frequently applied tool in the field of medical research, and it is a method that allows a good collection of data from patients [47]. Most of the studies conducted during the COVID-19 pandemic in the field of dentistry were represented by questionnaires sent via e-mail [48], online and on paper [49], or only on paper [50]. We chose to apply the questionnaires on paper in order to be able to clarify any questions that patients may have had while completing the form, without in any way influencing their answers. It also allowed us to apply them only to orthodontic patients.

Other studies that investigated the attitudes of dental or orthodontic patients toward protective face mask wearing have not been identified. For this matter, we consider this study to add information to the scientific literature. Nonetheless, this study has some limitations. First of all, it was applied only in the city of Oradea, Romania, and did not investigate children from other cities in Romania. The questionnaire is relatively short because we did not want children or adolescents to not be interested in completing it. More questions investigating other issues related to the COVID-19 pandemic could be added. An online questionnaire could be beneficial because it could allow other cities from Romania to be investigated, as well.

## 5. Conclusions

Face mask wearing is generally well tolerated by the Romanian orthodontic patients, living in the city of Oradea, with ages between 8 and 11.9 years. Most children were not stressed about the compulsoriness of face mask wearing and did not consider interrupting the orthodontic treatment due to the COVID-19 pandemic. Various differences were identified between children and adolescents, regarding the investigated items. As such, children stated significantly more often that they wanted to continue wearing a face mask, despite the fact that it covered the orthodontic appliance, while teenagers said significantly more often that they did not want to wear a face mask. Children also stated significantly more often that they wanted to correct the position of their teeth despite the fact that they had to wear a protective face mask that covered the orthodontic appliance, while teenagers stated significantly more often that they did not want to correct the position of their teeth while wearing a face mask. For most children, face masks do not seem to be a factor that discourages them to undergo an orthodontic treatment.

## Figures and Tables

**Table 1:** Distribution according to gender and living environment.

**Study group** **(children)**	**Gender**	
	Female (%, n)	Male (%, n)
	53.1% (n = 136)	46.9% (n = 120)
	**Living environment**	
	Rural (%, n)	Urban (%, n)
	32.8% (n = 84)	67.2% (n = 172)
**Control group** **(adolescents)** [26]	**Gender**	
	Female (%, n)	Male (%, n)
	62.5% (n = 173)	37.5% (n = 104)
	**Living environment**	
	Rural (%, n)	Urban (%, n)
	33.6% (n = 93)	66.4% (n = 184)

%—Percentage; n—number.

**Table 2:** Distribution of the patients according to the answers provided.

**Children (SG)**	**5 options Items**					
	**Answer** **(No., %)**	**Never**	**Rarely**	**Occasionally**	**Frequently**	**Very** **Frequently**
	Are you worried that wearing a protective face mask will hide your appliance? (Item 1)					
		103 (40.2%)	74 (28.9%)	43 (16.8%)	32 (12.5%)	4 (1.6%)
	Does the compulsoriness of wearing a protective face mask affect your desire to undergo the orthodontic treatment, given the fact that it covers your appliance? (Item 2)					
		95 (37.1%)	81 (31.6%)	54 (21.1%)	25 (9.8%)	1 (0.4%)
	Were you affected by the suspension of dental offices’ activity as a patient undergoing an orthodontic treatment? (Item 3)					
		122 (47.7%)	24 (9.4%)	22 (8.6%)	68 (26.6%)	20 (7.8%)
	Were you worried that you won’t be able to continue the orthodontic treatment due to the COVID-19 pandemic? (Item 4)					
		50 (19.5%)	62 (24.2%)	66 (25.8%)	42 (16.4%)	36 (14.1%)
	Do you consider that wearing a face mask that hides your orthodontic appliance causes you stress? (Item 8)					
		83 (32.4%)	24 (9.4%)	117 (45.7%)	30 (11.7%)	2 (0.8%)
	**3 options Items**					
		**No**		**Maybe**	**Yes**	
	Did you consider interrupting the orthodontic treatment because of the COVID-19 pandemic? (Item 5)					
		136 (53.1%)		69 (27%)	51 (19.9%)	
	Are you happy that you have to wear a face mask during the orthodontic treatment, considering the fact that it covers your appliance? (Item 6)					
		114 (44.5%)		108 (42.2%)	34 (13.3%)	
	Do you want face masks to continue being mandatory, given the fact that they cover your appliance? (Item 7)					
		126 (49.2%)		54 (21.1%)	76 (29.7%)	
	Do you still want to continue with the orthodontic treatment while wearing a face mask, even though your orthodontic appliance is not visible? (Item 9)					
		34 (13.3%)		44 (17.2%)	178 (69.5%)	
**Adolescents (CG)** [26]	**5 options Items**					
	**Answer** **(No., %)**	**Never**	**Rarely**	**Occasionally**	**Frequently**	**Very** **Frequently**
	Are you worried that wearing a protective face mask will hide your braces? (Item 1)					
		137 (49.5%)	74 (26.7%)	21 (7.6%)	26 (9.4%)	19 (6.9%)
	Does the compulsoriness of wearing a protective face mask affect your desire to undergo the orthodontic treatment, giving the fact that it covers your braces? (Item 2)					
		143 (51.6%)	72 (26%)	29 (10.5%)	25 (9%)	8 (2.9%)
	Were you affected by the suspension of dental offices’ activity, as a patient undergoing an orthodontic treatment with fixed appliances? (Item 3)					
		130 (46.9%)	44 (15.9%)	33 (11.9%)	39 (14.1%)	31 (11.2%)
	Were you worried that you won´t be able to continue the orthodontic treatment due to the COVID-19 pandemic? (Item 4)					
		66 (23.8%)	69 (24.9%)	64 (23.1%)	40 (14.4%)	38 (13.7%)
	Do you consider that wearing a face mask that hides your orthodontic appliance causes you stress? (Item 8)					
		143 (51.6%)	47 (17%)	63 (22.7%)	14 (5.1%)	10 (3.6%)
	**3 options Items**					
		**No**		**Maybe**	**Yes**	
	Did you consider interrupting the orthodontic treatment because of the COVID-19 pandemic? (Item 5)					
		173 (62.5%)		78 (28.2%)	26 (9.4%)	
	Are you happy that you have to wear a face mask during the orthodontic treatment, considering the fact that it covers your braces? (Item 6)					
		190 (68.6%)		63 (22.7%)	24 (8.7%)	
	Do you want face masks to continue being mandatory, given the fact that they cover your braces? (Item 7)					
		144 (52%)		102 (36.8%)	31 (11.2%)	
	Do you still want to continue with the orthodontic treatment while wearing a face mask even though your orthodontic appliance is not visible? (Item 9)					
		65 (23.5%)		30 (10.8%)	182 (65.7%)	

SG—Study Group; CG—Control Group; No.—Number; %—Percentage.

**Table 3:** Correlations between different items.

Correlations	*p* *
Item 1 Score (*p* < 0.001 **) × Item 2 Score (*p* < 0.001 **)	<0.001, R = 0.286
Item 1 Score (*p* < 0.001 **) × Item 8 Score (*p* < 0.001 **)	0.002, R = −0.196
Item 2 Score (*p* < 0.001 **) × Item 3 Score (*p* < 0.001 **)	0.011, R = −0.159
Item 3 Score (*p* < 0.001 **) × Item 4 Score (*p* < 0.001 **)	0.005, R = 0.174

* Spearman’s rho Correlation Coefficient, ** Shapiro-Wilk Test.

**Table 4:** Comparison of answers provided for various items in relation to respondents’ gender and living environment.

Variable	Mean Value ± SD	Median (IQR)	Medium Rank	*p* *
**Gender**				
**Item 4**				
Female (*p* < 0.001 **)	2.66 ± 1.295	2.5 (2–4)	120.03	0.046
Male (*p* < 0.001 **)	2.98 ± 1.316	3 (2–4)	138.10	
**Living environment**				
**Item 1**				
Rural (*p* < 0.001 **)	2.27 ± 1.068	2 (1–3)	144.12	0.013
Urban (*p* < 0.001 **)	1.96 ± 1.105	2 (1–2.75)	120.87	
**Item 4**				
Rural (*p* < 0.001 **)	3.1 ± 1.314	3 (2–4)	144.29	0.015
Urban (*p* < 0.001 **)	2.67 ± 1.293	3 (2–3)	120.79	

SD—Standard Deviation; IQR—Interquartile range; * Mann-Whitney U Test, ** Shapiro-Wilk Test.

**Table 5:** Comparisons between various items.

Comparison	Answer	Mean Value ± SD	Median (IQR)	Medium Rank	*p* *
**Item 3 and Item 5**	No (*p* < 0.001 **)	2.36 ± 1.47	2 (1–4)	127.18	0.003
	Maybe (*p* = 0.001 **)	2.77 ± 2.18	3 (1–4)	148.56	
	Yes (*p* < 0.001 **)	1.88 ± 1.38	1 (1–4)	104.89	
**Item 3 and Item 7**	No (*p* < 0.001 **)	2.27 ± 1.57	1 (1–4)	122.94	0.001
	Maybe (*p* < 0.001 **)	3.04 ± 1.24	3 (2–4)	158.61	
	Yes (*p* < 0.001 **)	2.08 ± 1.35	1 (1–3)	116.32	

SD—standard deviation; IQR—interquartile range; * Kruskal-Wallis H test, ** Shapiro-Wilk test.

**Table 6:** Comparison between children (study group) and adolescents (control group) for Likert-type scale items.

Group	Mean Value ± SD	Median (IQR)	Medium Rank	*p* *
**Item 1**				
Children (*p* < 0.001 **)	2.06 ± 1.101	2 (1–3)	266.26	0.910
Adolescents (*p* < 0.001 **)	2.17 ± 1.317	2 (1–3)	267.69	
**Item 2**				
Children (*p* < 0.001 **)	2.15 ± 1.046	2 (1–3)	280.18	0.043
Adolescents (*p* < 0.001 **)	2.01 ± 1.184	1 (1–3)	254.82	
**Item 3**				
Children (*p* < 0.001 **)	2.38 ± 1.483	2 (1–4)	270.00	0.645
Adolescents (*p* < 0.001 **)	2.31 ± 1.453	2 (1–4)	264.22	
**Item 4**				
Children (*p* < 0.001 **)	2.8 ± 1.316	3 (2–4)	272.26	0.438
Adolescents (*p* < 0.001 **)	2.71 ± 1.342	3 (2–4)	262.14	
**Item 8**				
Children (*p* < 0.001 **)	2.03 ± 0.98	2 (1–2)	285.54	0.004
Adolescents (*p* < 0.001 **)	1.86 ± 1.095	1 (1–3)	249.86	

SD—standard deviation; IQR—interquartile range; * Mann-Whitney U test, ** Shapiro-Wilk test.

**Table 7:** Comparison between children (study group) and adolescents (control group) for 3 options items.

Group	No	Maybe	Yes	*p* *
**Item 5**				
Children	136 (44%)	69 (46.9%)	51 (66.2%)	0.002
Adolescents	173 (56%)	78 (53.1%)	26 (33.8%)	
**Item 6**				
Children	114 (37.5%)	108 (63.2%)	34 (58.6%)	<0.001
Adolescents	190 (62.5%)	63 (36.8%)	24 (41.4%)	
**Item 7**				
Children	126 (46.7%)	54 (34.6%)	76 (31%)	<0.001
Adolescents	144 (53.3%)	102 (65.4%)	31 (29%)	
**Item 9**				
Children	34 (34.3%)	44 (59.5%)	178 (49.4%)	0.003
Adolescents	65 (65.7%)	30 (40.5%)	182 (50.6%)	

* Fisher’s exact test.

## Data Availability

The data presented in this study are available on request from the corresponding authors. The data are not publicly available due to privacy reasons.
